# Impact of Impedance Planimetry on Optimized Therapeutic Assessment in Complex Benign Esophageal Stenosis – A Prospective, Randomized Trial

**DOI:** 10.1055/a-2986-8685

**Published:** 2026-11-06

**Authors:** Kathrin Steger, Niklas Sturm, Martin Wagner, Martin Müller, Thomas Seufferlein, Benjamin M Walter

**Affiliations:** 1 Endoscopic Research Unit 27197 Ulm University HospitalUlmBWGermany; 2 Department of Internal Medicine I 27197 Ulm University HospitalUlmBWGermany

**Keywords:** esophageal stenosis, dilatation, impedance planimetry, EndoFLIP

## Abstract

**Background:**

Accurate evaluation of complex benign esophageal stenosis is essential for successful endoscopic dilation, yet conventional methods are either subjective or involve radiation exposure. This study investigated whether impedance planimetry (EndoFLIP) optimizes stricture geometry assessment compared with conventional nonradiographic methods and whether it improves patient outcomes.

**Patients and Methods:**

Totally 22 adult patients with symptomatic benign esophageal strictures were allocated to either a planimetry group or a control group. In the planimetry group, FLIP measurements were obtained before and after dilation in addition to standard endoscopic evaluation, whereas the control group underwent conventional assessment alone. Symptoms and quality of life were evaluated using the SWAL-QOL questionnaire pre- and 14 days postprocedure.

**Results:**

EndoFLIP measurements revealed significant increases in diameter, cross-sectional area, and distensibility following dilation. Compared with FLIP, stricture length was underestimated endoscopically by an average of 14.4 mm and post-therapeutic diameter was overestimated by 1.2 mm based on the final dilator size. G-SWAL-QOL total scores improved in both groups, whereas symptom scores and tolerance of solid food improved significantly in the planimetry group only.

**Conclusion:**

The use of impedance planimetry enabled a standardized and radiation-free assessment of complex benign esophageal stenosis and may contribute to optimizing the treatment of complex benign strictures of the upper gastrointestinal tract.

## Introduction

Complex benign esophageal stenosis poses a significant clinical challenge due to its frequently recurrent course, leading to persistent dysphagia that significantly compromises patients’ quality of life. 1 Potentially severe consequences, such as malnutrition and aspiration, further underscore the urgency for effective and safe therapeutic interventions. 2 Endoscopic dilation via bougienage or balloon dilation remains the cornerstone treatment for symptomatic esophageal strictures. 3 Strictures must be sufficiently expanded to alleviate symptoms while minimizing the risk of perforation or hemorrhage. The success of dilation depends on an accurate assessment of the stricture’s morphology, encompassing factors such as length, diameter, and etiology, as this guides both the selection of individually appropriate instruments and overall therapeutic strategy. 4 The selection of initial dilator size is directly based on the assessment of luminal geometry of the stenotic segment. 5 Currently, this is achieved through two established methods: endoscopic assessment and radiographic evaluation, if necessary. 5 6 Endoscopic estimation of luminal geometry is inherently subjective. Fluoroscopic imaging increases accuracy and objectivity but entails radiation exposure for patient and staff, particularly in frequently repeated interventions. To estimate the achieved dilation effect after bougienage, the defined diameters of the rigid bougie dilators serve as reference points. 4 The accuracy of this practice has, however, not yet been evaluated.

The Endoluminal Functional Lumen Imaging Probe (EndoFLIP, Medtronic, Minneapolis, MN, USA) offers a quantitative approach to characterizing the geometric and biomechanical properties of the esophagus, providing real-time measurements, and visualizing of luminal geometry via impedance planimetry. Data on the potential utility of EndoFLIP in the endoscopic management of adult benign esophageal stenosis remain limited.

This study aims to evaluate the clinical utility and feasibility of EndoFLIP in the management of adult complex benign esophageal stenosis. Specifically, we compared the assessment of stricture geometry using impedance planimetry with conventional methods and investigated whether the integration of EndoFLIP in the endoscopic management of esophageal stenosis improves patient outcomes.

## Patients and Methods

### Study Design

This prospective, randomized study enrolled patients aged ≥18 years with symptomatic complex benign esophageal strictures requiring endoscopic dilation. Complex strictures were defined by a length of ≥2 cm, a diameter of ≤12 mm, or irregular geometry on endoscopic evaluation. Patients were eligible irrespective of etiology, interval since the causative event, and previous endoscopic treatment. Exclusion criteria were lack of patient consent, malignant origin of stenosis, and absence of an indication for endoscopic treatment. In cases of repeated treatment, each patient was included in the study only once to prevent statistical bias. The random allocation sequence was computer-generated (1:1 ratio) using block randomization. Patients were blinded to their group allocation. Blinding of endoscopists was not feasible as they had to perform the intervention. Written informed consent was obtained from all participants, and the study was approved by the institutional review board and ethics committee of the University of Ulm (Germany). The trial adhered to the principles of the Declaration of Helsinki and was registered at clinicaltrials.gov (identifier: NCT07026357; 07/06/2022).

### Study Protocol

The study was conducted at the endoscopy unit of the University Hospital Ulm between December 2022 and March 2024. Patients were randomized into a planimetry group and a control group. In both groups, pre-therapeutic quality of life was assessed using the German version (G) of the SWAL-QOL questionnaire. All patients underwent endoscopic treatment with either bougienage or balloon dilation. In the control group, pre-therapeutic diameter and length of stenosis were assessed and estimated visually through the endoscope. Fluoroscopy was carried out at the discretion of the investigator and, where performed, supplemented the endoscopic assessment. After treatment, the achieved opening diameter was estimated conventionally based on the final bougie size. In the planimetry group, stricture length was estimated endoscopically, and pre-therapeutic FLIP measurements were performed by the endoscopist immediately prior to dilation. Dilator size and dilation tool were selected at the endoscopist’s discretion, informed by the FLIP-derived minimal diameter and stricture length. Post-therapeutic FLIP measurements were recorded for exploratory analysis. Data on the final bougie size representing conventional opening diameter assessment were available for this group as well. For all participants, quality of life (G-SWAL-QOL) was reassessed by telephone 14 days after treatment. Endoscopic follow-up was scheduled at approximately three months.

### Endoscopic Dilation

Esophagogastroduodenoscopy was conducted up to the stricture under propofol-based conscious sedation. If passage of a standard gastroscope with a 9.9 mm outer diameter (GIF-HQ190 or GIF-EZ1500, Olympus, Tokyo, Japan) was not possible, a pediatric gastroscope with a 5.4 mm diameter (GIF-H190N, Olympus, Tokyo, Japan) was used. Bougienage was performed using BougieCaps (Ovesco Endoscopy, Tübingen, Germany) or Savary-Gilliard bougies (Cook Medical, Bloomington, IN, USA) and balloon dilation with through-the-scope balloons of different sizes (Boston Scientific, Marlborough, MA, USA). Fluoroscopic guidance was employed when visualization was insufficient. In both groups, bougienage was performed incrementally, with the initial dilator size based on the assessment of stricture configuration; in general, two to three increments were carried out per session. The extent of dilation was determined by the endoscopist, guided by tissue resistance and the endoscopic appearance of the dilated segment, without reference to FLIP measurements. A postprocedure endoscopic check assessed potential adverse events.

### Impedance Planimetry

The EndoFLIP system (Medtronic, Minneapolis, MN, USA) was used to assess esophageal geometry during volume-controlled balloon distension. The catheter (EF-325N: 230 mm long, 3 mm diameter) features 16 impedance electrode pairs spaced 5 mm apart surrounded by an 8 cm long cylindrical balloon filled with a conductive solution. Impedance measurements determine the cross-sectional area at each electrode level. Assuming a circular lumen, the diameter is calculated. A real-time, color-coded topographic map visualizes the narrowed esophageal segment. An integrated solid-state pressure transducer measures intra-bag pressure, allowing for calculation of the distensibility index (DI), defined as the ratio of the minimal cross-sectional area to balloon pressure.

EndoFLIP measurements in the planimetry group were performed immediately before and after dilation therapy. After initialization and calibration, the catheter was positioned under endoscopic guidance with its center at the stricture’s narrowest point. The endoscope was removed, and the balloon was inflated with 40 mL of solution and the following parameters were recorded: minimal diameter (D _min_ , mm), length of stenosis (mm), balloon pressure (mmHg), minimal cross-sectional area (CSA _min_ , mm ^2^ ), and distensibility index (DI, mm ^2^ /mmHg). Each measurement was repeated three times, and the mean value was used for analysis.

### Symptom Assessment

Dysphagia-related symptoms and quality of life were assessed at baseline and 14 days postprocedure using the G-SWAL-QOL questionnaire, a validated German version of the SWAL-QOL questionnaire. 7 8 It consists of 48 items rated on a five-point Likert scale, covering various physical, psychological, and social impacts of dysphagia. A total score (0–100 points) was calculated by summing 30 item values and applying a linear transformation, with 0 indicating poor and 100 indicating excellent quality of life. A separate symptom battery (score range: 14–70 points with 70 indicating no symptoms) evaluated dysphagia-related symptom frequency and severity. Additionally, food consistency tolerated during the previous week (solid, soft or pureed food, or feeding via gastric tube) was recorded through the questionnaire.

### Outcome Measures

Baseline characteristics and demographic data were collected for all participants. For the between-group analysis, the primary outcome measure was the technical success rate, defined as sufficient dilation allowing endoscope passage after intervention. Secondary outcome measures included improvements in symptoms and quality of life, adverse events including perforation and hemorrhage, endoscopy time, sedation requirements, and the need for follow-up treatments. Within the planimetry group, baseline values and changes of FLIP measurements for esophageal stenosis were established and analyzed. FLIP parameters were compared between patients with and without the need for follow-up endoscopic treatment. A comparative analysis was conducted to assess the agreement between conventional estimates and FLIP measurements of stricture length and diameter. An intention-to-treat analysis was performed, including all data from patients who underwent randomization. For the G-SWAL-QOL questionnaires, only paired pre- and post-therapeutic responses were included; cases with missing post-therapeutic data were excluded from this analysis.

### Statistics

An 80% technical success rate for the conventional assessment was derived from prior institutional experience. Based on a noninferiority margin of 10%, a sample size of 60 patients in each group was calculated to demonstrate non-inferiority of impedance planimetry with a statistical power of 80% with a one-sided *α* -value of 0.025. Statistical tests were performed by using SPSS version 29 (IBM, Armonk, NY, USA). Descriptive statistics were computed for all variables to provide means and standard deviation or median and interquartile range for continuous variables and frequencies for categorical variables. Fisher’s exact test, Mann–Whitney *U* test, and Wilcoxon signed-rank test were used wherever applicable. A difference plot was employed to assess the agreement between conventional estimates and FLIP measurements of stricture geometry. A linear regression analysis evaluated proportional bias. A two-sided *P* -value < 0.05 was considered statistically significant.

## Results

A total of 22 patients were recruited and randomized into a planimetry and a control group. Age and sex were comparable between groups ( Table 1 ). The most common etiology was anastomotic stricture in the planimetry group and radiation-induced stricture in the control group. For strictures following surgery, radiotherapy, or ingestion, the median interval to intervention was 40.9 months (IQR: 14.4–86.8) and 44.8 months (IQR: 15.9–58.0), respectively. All strictures were fibrotic at the time of intervention with no signs of active inflammation. One patient in the planimetry group underwent balloon dilation, all other patients received bougienage. Bougienage was performed with BougieCaps except in one patient with the longest stricture (25 mm), who was treated with Savary-Gilliard bougies. Technical success of dilation was 100% in the planimetry group and 90.9% in the control group with one case of dilation failure. No adverse events such as perforation or persistent bleeding occurred in either group. There were no significant differences in endoscopy time, propofol dosage, and endoscopic follow-up treatments between groups ( Table 1 ).

**Table 1:** Patient characteristics and endoscopic results

	FLIP	Control	*p* -Value
	( *n* = 11)	( *n* = 11)	
**Age, median (IQR), years**	66 (10)	65 (26)	0.949
Range	31–79	35–90	
**Sex, n (%)**			0.748
Female	5 (45.5)	6 (54.5)	
Male	6 (54.5)	5 (45.5)	
**Etiology of stenosis, n (%)**			0.429
Anastomotic	5 (45.5)	2 (18.2)	
Radiation-induced	2 (18.2)	4 (36.4)	
Peptic	3 (27.3)	1 (9.1)	
Crohn’s disease	1 (9.1)	1 (9.1)	
Caustic	0	1 (9.1)	
Other	0	2 (18.2)	
**History of upper gastrointestinal surgery, n (%)**	6 (54.5)	4 (36.4)	0.670
**BMI, median (IQR), kg/m** ^2^	23.4 (10.2)	20.1 (6.7)	0.108
**Previous endoscopic treatment, n (%)**			0.478
Yes	6 (54.5)	8 (72.7)	
No	5 (45.5)	3 (27.3)	
**Dilation technique, n (%)**			1.000
Bougienage	10 (90.9)	11 (100)	
Balloon dilation	1 (9.1)	0	
**Technical success, n (%)**	11 (100)	10 (90.9)	1.000
**Use of fluoroscopy, n (%)**	0	3 (27.3)	0.300
**Adverse events, n**	0	0	1.000
**Endoscopy time, median (IQR), min**	28.0 (21.0)	37.0 (22.3)	0.705
**Propofol, median (IQR), mg**	270 (130)	230 (138)	0.282
**Diameter of stenosis before intervention, mean (s.d.), mm**	8.0 (1.6)	10.0 (2.3)	0.013
**Diameter of stenosis after intervention, mean (s.d.), mm**	11.2 (1.6)	13.2 (2.5)	0.013
**Length of stenosis, mean (s.d.), mm**	17.3 (3.4)	16.6 (14.4)	0.412
Range	15–25	1–50	
**Endoscopic follow-up, n (median interval, days)**	7 (144)	7 (116)	1.000
**Requirement of follow-up endoscopic treatment, n (%)**	3 (27.3)	4 (36.4)	1.000

IQR, interquartile range; s.d., standard deviation; BMI, body mass index.

Prior endoscopic treatment had been performed in six patients in the planimetry group and eight in the control group, with a shorter median interval since the last intervention in the control group (2.5 months, IQR: 1.7–5.3 vs. 6.1 months, IQR: 4.0–7.0, in the planimetry group). Follow-up endoscopic interventions were required in three patients in the planimetry group and four in the control group ( Table 1 ). Within the planimetry cohort, no FLIP-derived parameters were identified that predicted the need for follow-up endoscopic therapy.

### Symptoms and Quality of Life

A total of 11 G-SWAL-QOL questionnaires were available in the planimetry group and 8 in the control group, as two patients could not be reached, and one had died from a cause unrelated to the intervention. Before endoscopic therapy, food consistency tolerance was similar in both groups, with most patients restricted to pureed food ( Fig. 1 ). Following therapy, the proportion of patients able to consume solid food increased significantly in the planimetry group (9.1–63.6%, *P* = 0.014), whereas the control group exhibited a modest, nonsignificant increase (12.5–25.0%, *P* = 0.317, Fig. 1 ). Two patients in the planimetry group remained limited to pureed food after dilation compared with none in the control group.

**Fig. 1:**
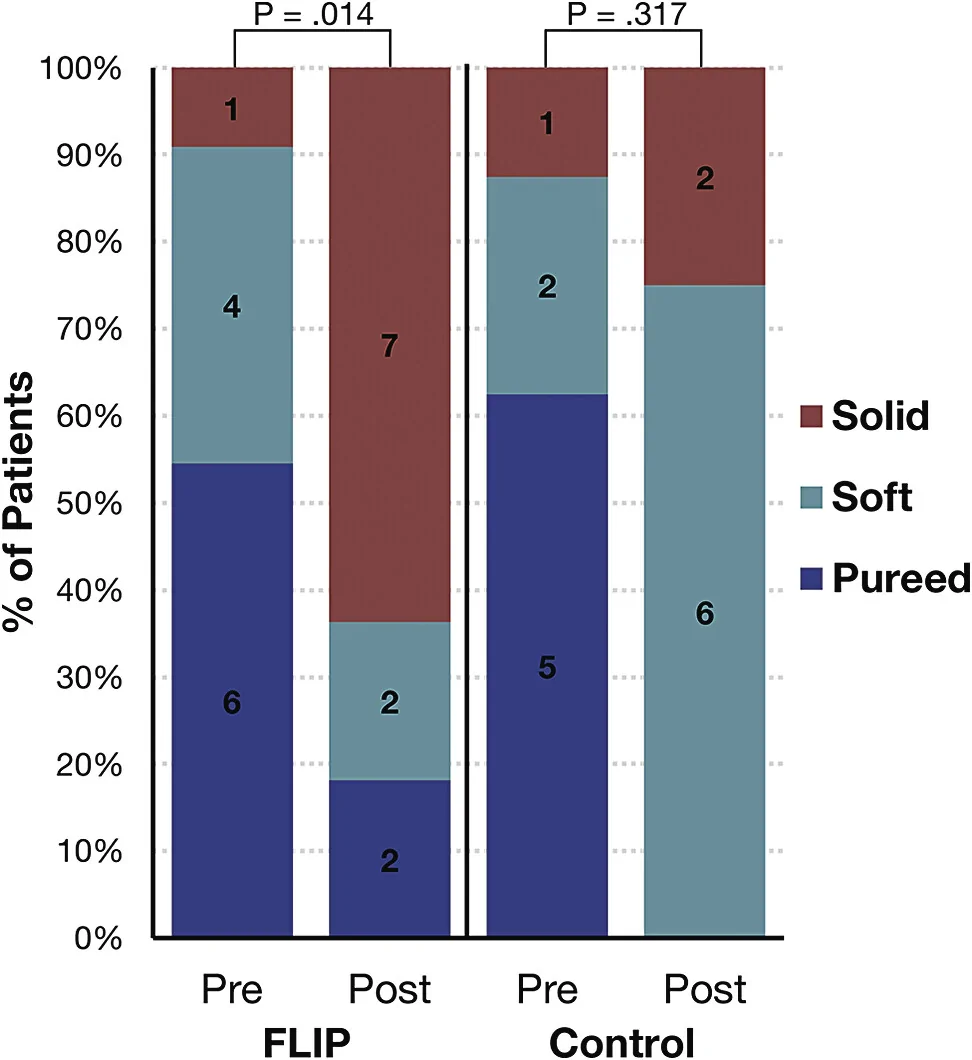
Food texture tolerance before and after endoscopic dilation, shown as number and percentage of patients. FLIP, planimetry group; Control, control group.

The control group demonstrated more severe and frequent symptoms as well as worse overall quality of life than the planimetry group. Symptom scores improved more markedly in the planimetry group, with a significant median symptom score increase from 56 to 61 points 14 days after dilation ( *P* = 0.005). The control group showed a nonsignificant median increase from 42 to 44 points ( *P* = 0.176). Total score increased in both groups, although to a greater extent in the planimetry group (planimetry group: 62.5–79.2, *P* = 0.008; control group: 45.0–52.1, *P* = 0.017).

### D _min_ , CSA _min_ , and DI Measurements Reflect Esophageal Stenosis and Dilation

EndoFLIP measurements were feasible and visualized the esophageal stricture geometry and its change after dilation ( Fig. 2 ). Mean minimal opening diameter (D _min_ ), minimal cross-sectional area (CSA _min_ ), and distensibility index (DI) increased significantly following stricture dilation ( Table 2 ).

**Fig. 2:**
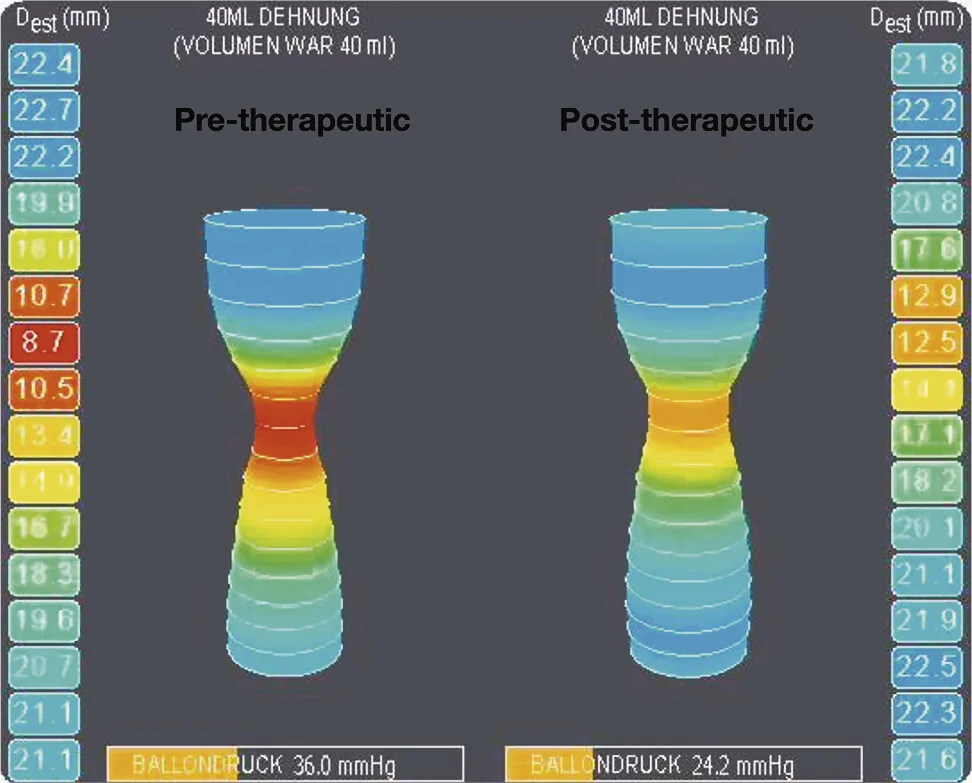
Pre-therapeutic and post-therapeutic EndoFLIP visualization of esophageal stenosis.

**Table 2:** Baseline values and changes of FLIP parameters during endoscopic intervention

	Pre-therapeutic, mean (s.d.)	Post-therapeutic, mean (s.d.)	*p* -Value
	( *n* = 11)	( *n* = 11)	
D _min_ (mm)	8.0 (1.6)	11.2 (1.6)	0.003
Length (mm)	17.3 (3.4)	17.3 (3.4)	1.000
Balloon pressure (mmHg)	53.1 (26.3)	46.8 (28.8)	0.182
CSA _min_ (mm ^2^ )	53.1 (18.0)	103.8 (30.7)	0.003
DI (mm ^2^ /mmHg)	1.2 (0.6)	2.6 (1.4)	0.004

s.d., standard deviation, D _min_ , minimal diameter, CSA _min_ , minimal cross-sectional area, DI, distensibility index.

The initial dilator diameter was chosen a mean of 1.7 mm above the pre-therapeutic FLIP-derived minimal opening diameter (range: 0–3.1 mm).

### Conventional Estimation and Impedance Planimetry Measurement of Stricture Geometry

The endoscopic estimation of stricture length prior to the first FLIP measurement averaged 3.1 mm ( *n* = 10). In the same patients, EndoFLIP subsequently determined a mean stricture length of 17.5 mm, revealing an average discrepancy of 14.4 mm in length assessment between endoscopy and impedance planimetry ( *P* = 0.004, 95% confidence interval −16.4 to −12.5 mm, Fig. 3 ). The discrepancy ranged from 10.0 to 20.0 mm ( Fig. 3 ). No indication of a proportional bias was observed in the scatter plot and regression analysis.

**Fig. 3:**
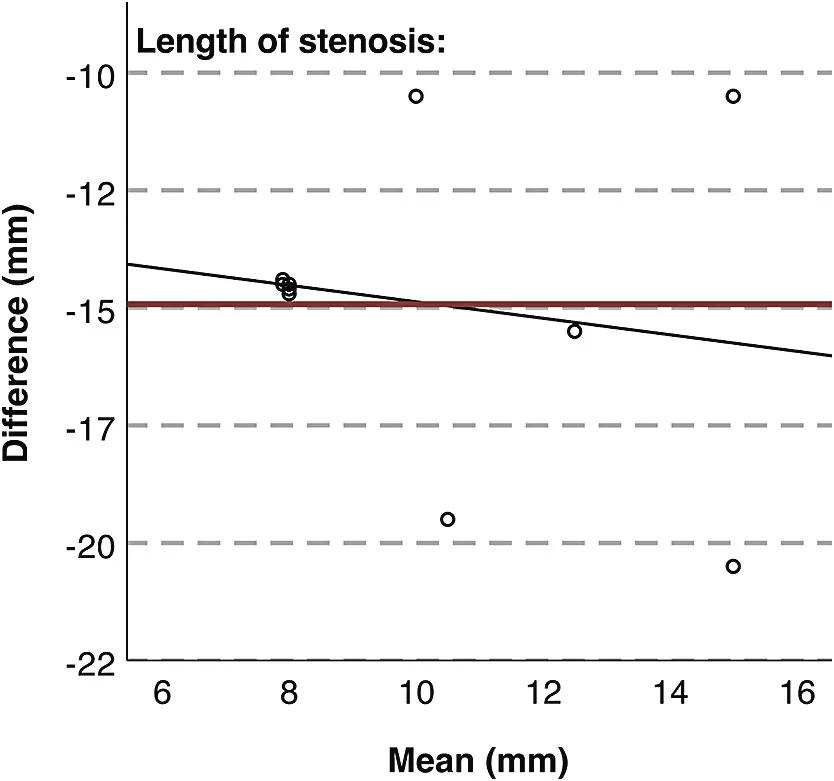
Differences between conventional endoscopic estimates and FLIP measurements of stricture length (endoscopic minus FLIP; negative values indicate underestimation by endoscopy), plotted against the mean of both methods. The red horizontal line represents the mean difference, the black line indicates the linear regression.

The mean minimal post-therapeutic diameter, as estimated based on the final bougie size, was 12.7 mm ( *n* = 10). Subsequent EndoFLIP measurements determined a mean minimal diameter of 11.5 mm, resulting in an average deviation of 1.2 mm between the two methods in the assessment of postdilation diameter ( *P* = 0.007, 95% confidence interval 0.4–1.8 mm, Fig. 4 ). Differences ranged between −0.1 and 3.4 mm ( Fig. 4 ). Discrepancies between the two methods increased with larger mean values, suggesting a proportional bias ( Fig. 4 ). Linear regression analysis showed a significant correlation between the differences and mean values (regression coefficient *B* = 0.43, *P* = 0.012), further indicating an increasingly systematic deviation in bougie-based diameter assessment at larger diameters.

**Fig. 4:**
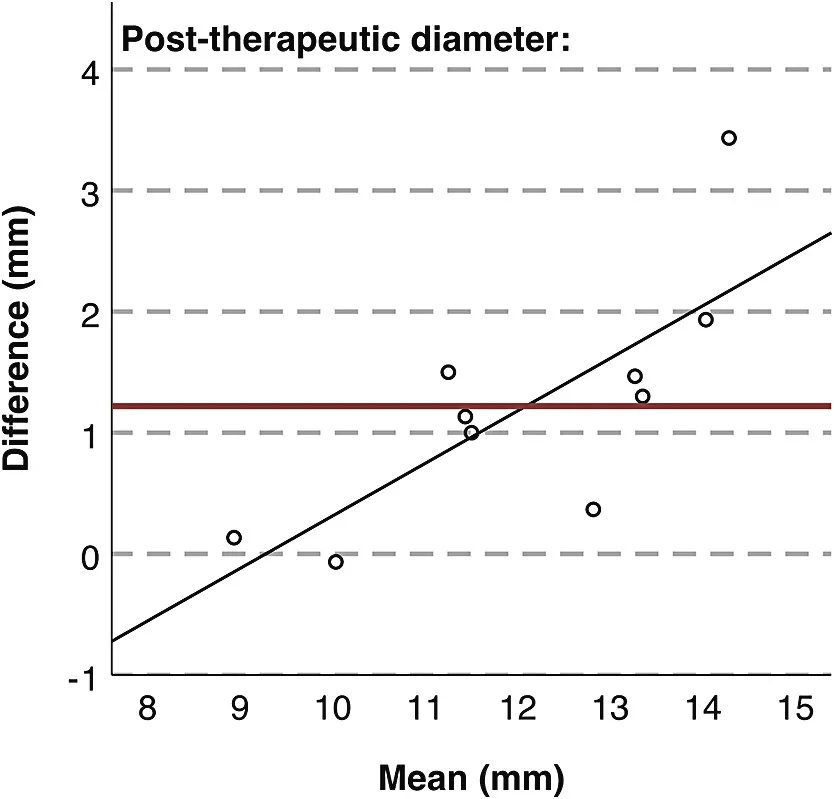
Differences between the final bougie size and FLIP measurements of post-therapeutic minimal diameter (bougie minus FLIP; positive values indicate overestimation by bougie size), plotted against the mean of both methods. The red horizontal line represents the mean difference, the black line indicates the linear regression.

## Discussion

This study is the first to demonstrate the feasibility of EndoFLIP for visualizing complex esophageal stricture geometry in adults. Our findings indicate that the conventional endoscopic assessment of stricture configuration is prone to systematic errors. Impedance planimetry shows promise as a radiation-free and standardized tool for more accurate stricture evaluation, potentially improving therapeutic decision-making and enhancing symptomatic outcomes.

The stenosis and dilation processes were consistently characterized by EndoFLIP measurements. FLIP has been reported to visualize strictures reliably in eosinophilic esophagitis and to be safely applicable in pediatric esophageal stenosis. 9 10 Our study extends these observations to the adult population, comparing stricture characterization and outcome parameters between EndoFLIP-assisted and conventional approaches.

Our data indicate that endoscopic evaluation systematically underestimates stricture length, with an average discrepancy exceeding 14 mm. This may result from a limited endoscopic perspective when viewing from above and during passage, leading to an under-recognition of the full extent of the stenotic segment beyond its narrowest visible point, whereas impedance planimetry captures the entire segment of reduced diameter. Furthermore, the data suggest that the dilation effect was overestimated when inferred from the largest bougie diameter advanced through the esophageal lumen, although the mean difference was modest at 1.2 mm. This could be attributed to the lumen not retaining the transient expansion achieved during bougie passage. Discrepancies were more pronounced at larger post-therapeutic diameters, and the underlying mechanism warrants further investigation. Target diameters associated with improved outcomes have been defined conventionally, typically ranging from at least 15 to over 16 mm. 5 18 19 If conventional assessment systematically overestimates the achieved diameter, such targets may need to be redefined using measured values, for which FLIP could provide a standardized basis.

Other studies have reported low sensitivity of endoscopy compared with esophagography for esophageal stricture detection. 11 12 Furthermore, interobserver variability in endoscopic assessment remains a concern due to its subjective nature. For complex strictures in which endoscopic visualization is inadequate, fluoroscopic guidance is recommended. 1 Nevertheless, several studies have stated that wire-guided dilation without fluoroscopy is comparably safe and effective. 13 14 15 For dilations performed solely under endoscopic visualization and wire guidance, radiation exposure is reduced; however, dilator selection then relies exclusively on endoscopic judgment of lumen morphology.

Our results indicate that impedance planimetry may offer a more precise assessment of esophageal stricture length and diameter than endoscopic evaluation; its potential as an objective diagnostic tool has already been described in gastric sleeve stenosis. 16 While fluoroscopy remains essential in select cases, EndoFLIP could serve as a radiation-free alternative for stricture configuration assessment. After dilation, FLIP’s real-time display of esophageal peristalsis also offers immediate functional feedback on swallow dynamics.

In this study, FLIP measurements provided an objective reference for initial dilator selection; in the absence of established criteria for the initial dilator diameter, the final choice remained at the endoscopist’s discretion. We postulate that the enhanced understanding of stricture geometry provided by EndoFLIP permits closer matching of initial dilator size to the luminal diameter and informs the choice of the dilation tool, taking stricture length into account. This may enable more targeted and risk-adapted therapy.

Our results did not demonstrate the superiority of either method with respect to technical success rates, the primary outcome measure, or adverse event rates, which may be due to small sample size. Differences were confined to symptomatic domains: the planimetry group showed a greater increase in the ability to consume solid food, the outcome most directly reflecting relief of dysphagia, despite similar baseline distributions. The same trend was seen in total and symptom scores, although the lower baseline scores in the control group and the shorter interval since the last endoscopic treatment, suggesting a more refractory course, limit the comparability of these changes.

Overall procedure duration was not significantly prolonged in the planimetry group, contrasting with a previous report of a median 6-minute increase from FLIP measurements. 17 This suggests that intervention- and patient-related factors exert a greater influence on endoscopy duration. The measurement itself was not timed separately, so its individual contribution cannot be quantified. Resource use, however, is not limited to procedure time: Impedance planimetry requires a one-time investment in the recording system and a single-use catheter for each procedure, whereas fluoroscopy may be reduced.

We acknowledge several limitations of this study. First, enrollment was restricted to a single center, so that the calculated sample size was not reached given the rarity of this condition. This limits generalizability, and the results should therefore be regarded as exploratory.

Second, broad eligibility criteria yielded a heterogeneous cohort of treatment-naive and pretreated patients, with anastomotic strictures predominating in the planimetry group and radiation-induced strictures in the control group. Etiology and previous treatment may affect the response to dilation, although the comparable interval since the causative event indicates strictures of similar maturity. Together with the lower baseline scores in the control group, this may have contributed to the observed differences in symptomatic improvement. Dilation technique was likewise not standardized; stratified analysis was not feasible for either.

Third, differences in evaluation methods limit direct comparisons of stricture geometry between groups. While consistently incorporating fluoroscopy in both arms would improve comparability, it would artificially influence diagnostic and therapeutic decisions in the control group, limiting the clinical applicability of the results as fluoroscopy is not always available in practice.

Finally, the validity of EndoFLIP was not specifically evaluated. Comparative studies with radiographic methods or phantom studies could provide further insights on the accuracy of FLIP. Moreover, the influence of different balloon filling volumes on cross-sectional areas requires further investigation. Despite these limitations, this is, to our knowledge, the first study to demonstrate the feasibility and potential add-on value of EndoFLIP in adult complex esophageal stenosis, providing preliminary insights that can inform the design of future studies.

## Conclusion

In conclusion, our study demonstrated that EndoFLIP provides a radiation-free and more objective evaluation of esophageal stricture geometry compared with conventional endoscopic assessment. This enhanced characterization may facilitate more targeted therapeutic decision-making and holds promise for improving symptomatic outcomes, although confirmation in larger cohorts is required. Our findings support the potential of impedance planimetry to standardize stricture evaluation while minimizing fluoroscopy and to optimize treatment strategies in adult esophageal stenosis.

## Data Availability

The underlying research data of this article are available from the corresponding author on reasonable request.

## Declaration of GenAI Use

The authors confirm that no Generative AI was used in the preparation of this manuscript.
